# The Value of a Seven-Autoantibody Panel Combined with the Mayo Model in the Differential Diagnosis of Pulmonary Nodules

**DOI:** 10.1155/2021/6677823

**Published:** 2021-02-20

**Authors:** Zhougui Ling, Jifei Chen, Zhongwei Wen, Xiaomou Wei, Rui Su, Zhenming Tang, Zhuojun Hu

**Affiliations:** ^1^Department of Pulmonary and Critical Care Medicine, The Fourth Affiliated Hospital of Guangxi Medical University, No. 1, Liushi Road, Liuzhou 545005, China; ^2^Clinical Laboratory, The Fourth Affiliated Hospital of Guangxi Medical University, No. 1, Liushi Road, Liuzhou 545005, China

## Abstract

**Background:**

Identifying malignant pulmonary nodules and detecting early-stage lung cancer (LC) could reduce mortality. This study investigated the clinical value of a seven-autoantibody (7-AAB) panel in combination with the Mayo model for the early detection of LC and distinguishing benign from malignant pulmonary nodules (MPNs).

**Methods:**

The concentrations of the elements of a 7-AAB panel were quantitated by enzyme-linked immunosorbent assay (ELISA) in 806 participants. The probability of MPNs was calculated using the Mayo predictive model. The performances of the 7-AAB panel and the Mayo model were analyzed by receiver operating characteristic (ROC) analyses, and the difference between groups was evaluated by chi-square tests (*χ*^2^).

**Results:**

The combined area under the ROC curve (AUC) for all 7 AABs was higher than that of a single one. The sensitivities of the 7-AAB panel were 67.5% in the stage I-II LC patients and 60.3% in the stage III-IV patients, with a specificity of 89.6% for the healthy controls and 83.1% for benign lung disease patients. The detection rate of the 7-AAB panel in the early-stage LC patients was higher than that of traditional tumor markers. The AUC of the 7-AAB panel in combination with the Mayo model was higher than that of the 7-AAB panel alone or the Mayo model alone in distinguishing MPN from benign nodules. For early-stage MPN, the sensitivity and specificity of the combination were 93.5% and 58.0%, respectively. For advanced-stage MPN, the sensitivity and specificity of the combination were 91.4% and 72.8%, respectively. The combination of the 7-AAB panel with the Mayo model significantly improved the detection rate of MPN, but the positive predictive value (PPV) and the specificity were not improved when compared with either the 7-AAB panel alone or the Mayo model alone.

**Conclusion:**

Our study confirmed the clinical value of the 7-AAB panel for the early detection of lung cancer and in combination with the Mayo model could be used to distinguish benign from malignant pulmonary nodules.

## 1. Introduction

Lung cancer (LC) remains the highest cause of cancer-related death for both sexes in the United States and worldwide [1, 2]. Early detection of LC and timely resection could reduce the mortality rate associated with this disease. Compared to chest radiography, annual low dose computed tomography (LDCT) screening is associated with a 20% reduction in LC mortality in high-risk individuals [3]. With the movement toward screening for LC at an early stage by LDCT or the widespread use of multidetector CT technology, an increasing number of pulmonary nodules (PNs) are being detected [4, 5, 6]. However, the drawbacks of LDCT screening, including with high rate of false-positive rate results (96.4%) to distinguish benign nodules from early-stage malignant cancer, not only lead to unnecessary follow-up toxic radiation scans and invasive follow-up procedures [3, 7] but also bring no benefit to the outcomes of small cell lung cancer (SCLC) patients [8]. Due to the small lesion volume and the lack of specific CT imaging features for distinguishing between benign and malignant nodules, it has long been challenging for clinicians to identify malignant pulmonary nodules (MPNs) from benign pulmonary nodules (BPNs). Most clinicians diagnose PNs mainly based on their personal clinical experience or specific CT imaging features, which may be subjective. A mathematical predictive model is an objective evaluation method based on statistics; therefore, one could be expected to help physicians distinguish benign from malignant nodules, avoiding subjective and one-sided judgments [9]. The Mayo Clinic model, published in 1997, is the first and still widely used model focusing on solitary pulmonary nodules (SPNs). It includes six variables (age, smoking history, cancer history, nodule diameter, location of the nodule, and speculation), with an area under curve (AUC) of 0.832 for predicting malignancy [10]. However, the Mayo model may underestimate the probability of malignancy in low-risk patients or have poor calibration in patients referred for surgical evaluation [11, 12]. Therefore, another adjunctive test is extremely essential to improve differentiating benign from malignant nodules and reduce the false-positive rate.

Tumor-associated autoantibodies (AABs), formulated from tumor-associated antigens (TAAs) captured by the humoral immune system, may be used to identify individuals with early lung cancer or distinguish MPNs from BPNs [13]. AABs can be detected before the disease becomes symptomatic and may even be found up to 5 years before CT is able to identify the tumor [14]. For the heterogeneity of single antigen expression, many studies have focused on panels of autoantibodies as blood biomarkers to diagnose early LC or to distinguish benign from malignant nodules, but the diagnostic accuracy has been inconsistent [13, 15–20]. Our previous meta-analysis showed that the sensitivities of two panels, one using 7 AABs and the other 6 AABs, were 40% and 29.7% in the early detection of LC, while their specificities were 91% and 87%, respectively. [21] Recently, a seven-AAB panel (p53, PGP9.5, SOX2, GAGE7, GBU4-5, CAGE, and MAGEA1) was developed and commoditized in China, which is mainly used as a new biomarker in the early diagnosis of lung cancer, with a sensitivity range from 56.5% to 62% and a specificity range from 90% to 91.6% in the detection of early-stage LC. When combined with CT, the diagnostic yield could be improved in patients presenting with ground-glass nodules (GGNs) and/or solid nodules [13, 22, 23]. However, none of the current panels showed enough sensitivity to make them ideal serum biomarkers for the early detection of LC. In the present study, we not only validated the clinical value of the 7-AAB panel in the early detection of LC but also evaluated the value of the utility of the 7-AAB panel in combination with the Mayo model to distinguish between benign and malignant nodules.

## 2. Materials and Methods

### 2.1. Patients and Blood Samples

This study was a diagnostic cohort test (registration number: ChiCTR-DDD-17010378) approved by the ethics committee of the Fourth Affiliated Hospital of Guangxi Medical University (number KY2016208). Blood samples were collected from 806 participants (Tables 1 and 2), which included patients with histopathologically confirmed LC, benign pulmonary disease (BLD) and pulmonary nodules (PNs) as well as healthy controls, in our hospital from January 2017 to May 2019. Informed written consent was obtained from each participant. LC or MPN was defined based on CT scans and verified by histopathology according to the World Health Organization Classification of Tumors [24]. The diagnosis of BLD was established by clinical data and CT scans. Pulmonary nodules were diagnosed by CT scans, and follow-up was performed strictly according to the Clinical Practice Consensus Guidelines [25]. The patients' blood samples were collected at initial diagnosis. None of the LC patients had received preoperative chemotherapy or radiotherapy. The healthy controls were recruited during health examinations, and none showed evidence of malignancy. A PN is diagnosed clinically as a benign etiology if it accords with one of the following: (1) definitive pathologic diagnosis, (2) radiographic resolution, or (3) no evidence of growth according to CT scan for 1 year [26]. Supernatants were obtained from blood samples through centrifugation at 3,000 g for 15 minutes at 4°C and were immediately subpackaged and then stored at -80°C until analyzed.

### 2.2. Quantitation of AABs or TAAs in Serum Samples

The serum concentrations of the 7-AAB panel (p53, GAGE7, PGP9.5, CAGE, MAGEA1, SOX2, and GBU4-5) were quantitated by an enzyme-linked immunosorbent assay (ELISA), and a commercial AABs assay (Cancer Probe Biological Technology Co., Ltd, Hangzhou, China) was conducted according to the manufacturer's recommendations and measured as previously described [13, 23]. Briefly, the samples and kit components were equilibrated to room temperature and diluted with phosphate-buffered saline (PBS) [1 : 109]. Then, 50 *μ*L of diluted serum samples and standards was added to appropriate wells and incubated for 1 h. After washing the plate 3 times, 50 *μ*L of diluted secondary antibody anti-human IgG HRP was added to each well to bind the autoantibodies. The plate was washed 3 times and incubated for half an hour. The substrate was added, and the color development reaction was terminated after 15 min with 50 *μ*L of stop solution. The OD at 450 nm was read using a spectrophotometer within 30 min. Each sample was tested in duplicate. We applied preset commercial cutoff values that had the maximum sensitivity with a fixed specificity of 90% using a Monte Carlo direct search method [27].

The serum concentrations of traditional TAA markers (CYFR21, CEA, NSE, and SCC) were quantitated by an electrochemiluminescent immunoassay. All assays were performed according to instrument and reagent specifications, and cutoff values were set according to the manufacturers' recommendations. The laboratory technicians were blinded to the patient's identity, and the results were analyzed blindly by another investigator.

### 2.3. Mayo Model for Predicting Malignancy

The probability of malignancy of the PNs was calculated using the Mayo predictive model, which is defined by the following equations: probability (*P*) = *e*^*x*^/(1 + *e*^*x*^), *x* = −6.8272 + (0.0391 × age) + (0.7917 × smoking history) + (1.3388 × cancer history) + (0.1274 × diameter) + (1.0407 × spiculation) + (0.7838 × upper lobe), where *e* is the base of the natural logarithm, and the smoking history, cancer history, spiculation, and upper lobe variables can be either 1 for yes or 0 for no. Diameter indicates the largest nodule measurement (in mm) reported on initial chest radiograph or CT scan [28]. According to the American College of Chest Physicians (ACCP) guidelines, when the *P* is <5%, watchful waiting is preferred. When the *P* is 5% to 65%, needle biopsy is preferred. When the *P* is >65%, surgery is preferred [29].

### 2.4. Statistical Analysis

The data were described as the means ± standard deviations (SDs) for continuous variables and frequency and percentage for categorical variables. The differences of the seven AABs in the serum levels among the groups were compared using nonparametric tests (Mann–Whitney *U*-test). Sensitivity and specificity were calculated according to the cutoff value. To confirm the sensitivity and specificity results, receiver operating characteristic (ROC) curves were constructed, and the area under the ROC curve (AUC) was calculated. Chi-square tests (*χ*^2^) were used to evaluate the difference between 2 groups. A 2-sided *P* value < 0.05 indicated statistical significance. All statistical analyses were carried out using the SPSS 22.0 (SPSS Inc., Chicago, IL, USA), and GraphPad Prism 5.0 software (GraphPad Software Inc., San Diego, CA, USA) was used for image editing.

## 3. Results

### 3.1. Patients' Characteristics

A total of 806 participants (193 + 135 + 118 + 360) were included in the study. A total of 193 LC patients with different disease stages (153 with non-small-cell lung cancer (NSCLC) and 40 with SCLC), 118 patients with benign lung diseases, and 135 healthy controls were included. There were more LC patients in the advanced-stage (III-IV) (60.1%) than in the early stage (I-II) (39.9%). The etiologic diagnoses of the BLD group included bronchitis, community-acquired pneumonia (CAP), chronic obstructive pulmonary disease (COPD), obstructive sleep apnea syndrome (OSAS), cough-variant asthma (CVA), bronchiectasis, parapneumonic effusion, and pulmonary tuberculosis. The clinical characteristics of the study population are summarized in Table 1.

After screening with LDCT in the high-risk population with a history of heavy tobacco usage, 360 PN patients (including 162 patients with BPN and 198 with undetermined nodules) were included to test the utility of the 7-AAB panel and the Mayo model in the differential diagnosis of PNs. The major clinical characteristics of this population are summarized in Table 2.

### 3.2. The Reactivity Performance of the 7 AABs in Lung Cancer Patients and Healthy Controls

**Figure 1:**
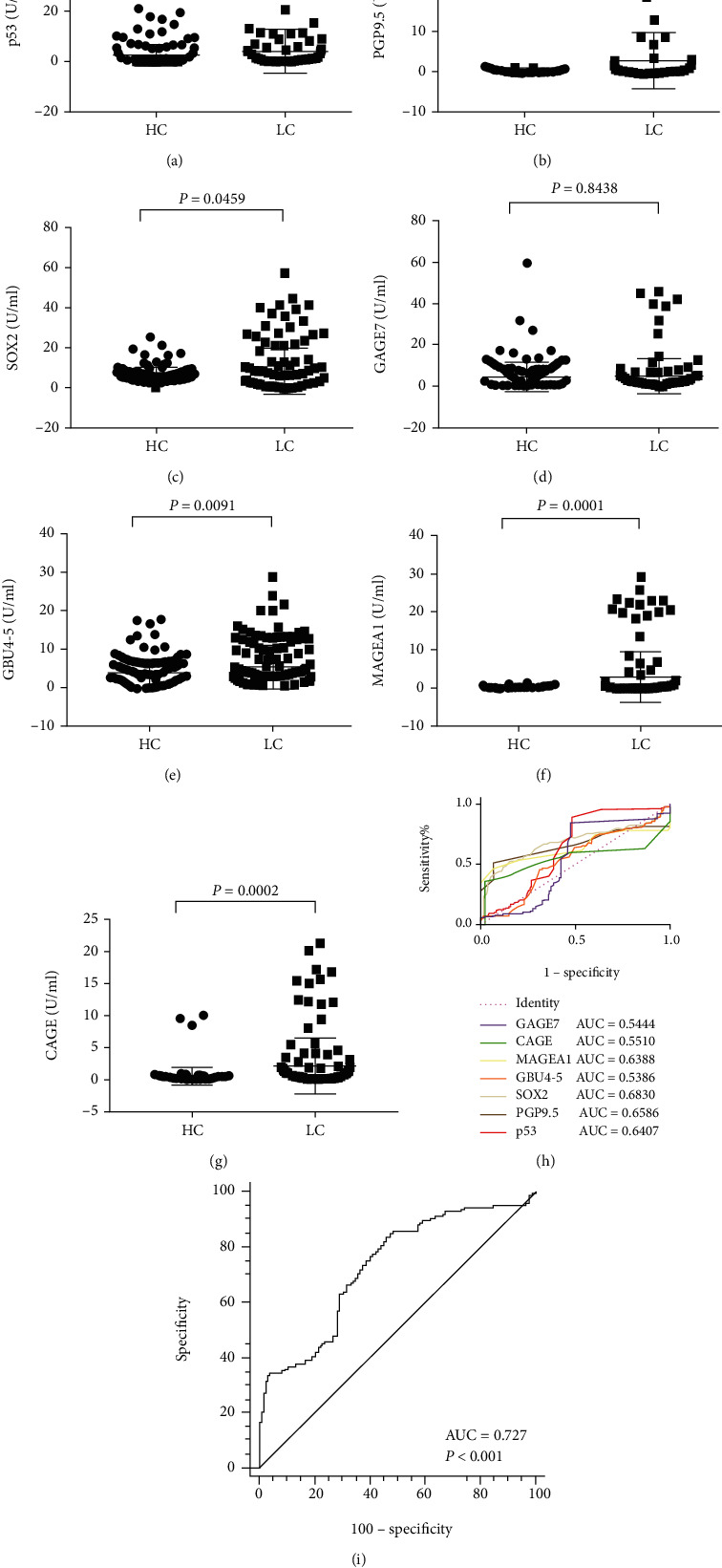
Concentration and the area under the curve (AUC) of each autoantibody between lung cancer (LC) cases and health controls (HC). (a) p53; (b) PGP9.5; (c) SOX2; (d) GAGE7; (e) GBU4-5; (f) MAGEA1; (g) CAGE; (h) AUCs for each autoantibody; (i) combined AUC for the 7-AAB panel.

To determine the reactivity of the panel of 7 AABs, we measured the concentrations of the 7 AABs in 193 LC patients and 135 healthy controls. The results showed that the serum AAB concentrations of p53, PGP9.5, SOX2, GBU4-5, MAGEA1, and CAGE in the LC patients were markedly higher than those in the healthy controls (*P* = 0.042, *P* < 0.001, *P* = 0.046, *P* < 0.001, *P* < 0.001, and *P* < 0.001, respectively), but the expression level of GAGE7 in the LC group was similar to that of the healthy group (*P* = 0.844) (Figures 1(a)–1(g)). Although most of the AABs except PGP9.5 demonstrated good discriminative ability between lung cancer and healthy controls, the AUCs of the single AAB showed poor diagnostic efficacy (all*P* < 0.7). However, the combined AUC for all 7 AABs improved to 0.727, which indicated good diagnostic efficacy (Figures 1(h) and 1(i)).

### 3.3. The Diagnostic Value of the 7-AAB Panel for Lung Cancer

**Figure 2:**
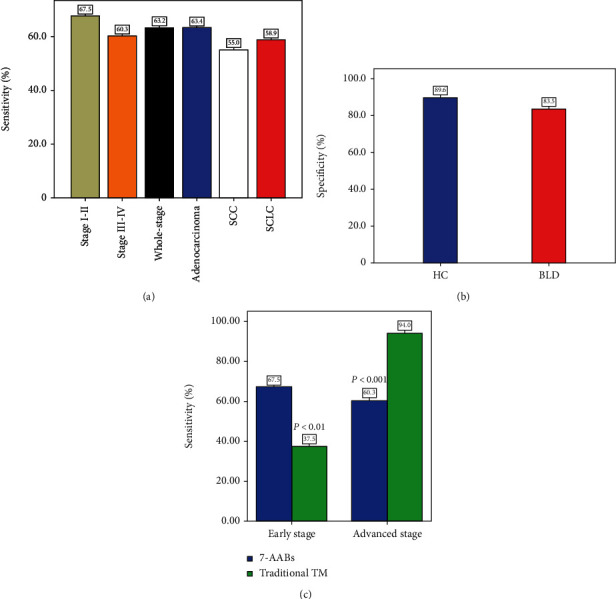
Diagnostic performance of the 7-AAB panel in lung cancer (LC) patients. SCC: squamous cell carcinoma; SCLC: small-cell lung cancer; TM: tumor markers; HC: healthy controls; BLD: benign pulmonary disease. (a) Sensitivities of the 7-AAB panel in different disease stages and histological types; (b) specificities of the 7-AAB panel in HC and BLD patients; (c) comparison of the 7-AAB panel and traditional tumor markers in LC patients.

Using the commercial assay cutoffs, positivity is defined as having an elevated AAB assay signal to any one of the antigens in the 7-AAB panel. The predictive power of this 7-AAB panel for the diagnosis of whole-stage lung cancer revealed a sensitivity of 63.2% (122/193), with a specificity of 89.6% (121/135) in the healthy controls and 83.1% (98/118) in the BLD group (Table 1, Figures 2(a) and 2(b)).

We also conducted subgroup analyses to investigate the diagnostic value of the 7-AAB panel in patients with different disease stages and histological types. The sensitivities were 67.5% (52/77) in stages I-II of the disease, 60.3% (70/116) in stages III-IV of the disease, 55.0% (22/40) in SCLS, 63.4% (71/112) in adenocarcinoma, and 58.9% (23/39) in squamous cell carcinoma (Figure 2(a)).

Moreover, we simultaneously measured the serum 7-AAB panel and the combination of traditional tumor markers (CYFR21, CEA, NSE, and SCC) in the same patient. The results showed the sensitivity values of the 7-AAB panel in the early-stage LC patients were higher than those of the traditional tumor markers (67.5% vs. 37.5%, *P* < 0.01) but were lower those in the late-stage LC patients (60.3% vs. 94.0%, *P* < 0.001) (Figure 2(c)).

### 3.4. The Performance of the 7-AAB Panel in Combination with the Mayo Model in Distinguishing Benign from Early-Stage MPN

The 7-AAB test and the Mayo prediction model were assessed for the presence of PNs. After excluding 198 participants with undetermined nodules, 355 PN patients were included in the analysis. Among them, 116 patients were pathologically diagnosed with advanced stage (III-IV) MPNs, 77 with early-stage (I-II) MPNs, and 162 with benign pulmonary nodules (BPNs).

**Figure 3:**
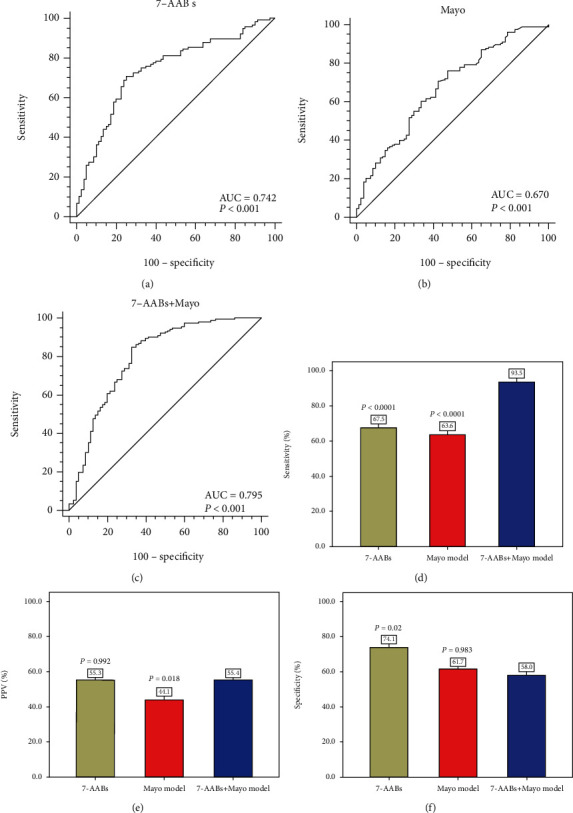
Diagnostic performance of the 7-AAB panel and the Mayo model between benign pulmonary nodules (BPN) and malignant pulmonary nodules (MPN) with early-stage. (a) The AUC of the 7-AAB panel; (b) the AUC of the Mayo model; (c) the AUC of the 7-AAB panel combination with the Mayo model; (d) sensitivity in MPN patients with early stage; (e) positive predictive values (PPVs) in MPN patients with early stage; (f) specificity in BPN patients.

First, we evaluated the diagnostic value of the 7-AAB panel and the Mayo model to distinguish early-stage (I-II) MPN patients from the BPN controls. The rates of nodule sizes < 8 mm and Mayo malignancy probability < 5% in the patients with BPN were greater than those of the early-stage MPN patients (*P* < 0.0001), but the rates of nodule sizes > 8 mm and Mayo malignancy probability > 5% were greater in the MPN patients than in the BPN controls. The positive rates of early-stage malignant nodules were higher than those of benign nodules for both the 7-AAB panel (67.5% vs. 25.9%; *P* < 0.0001) and the Mayo model with probabilities between 5 and 65% (54.5% vs. 34.6%; *P* < 0.001) (Table 2). The AUCs (95% CI) for the 7-AAB panel, the Mayo model, and the 7-AAB panel+the Mayo model between the two groups were as follows: 0.742 (0.674-0.801), 0.670 (0.605-0.730), and 0.795 (0.738-0.845), respectively; the 7-AAB panel+the Mayo model showed improved diagnostic accuracy (Figures 3(a)–3(c)). When the cutoff value of malignancy probability was set at >5%, the detection rate was 63.6%. The 7-AAB panel in combination with the Mayo model significantly improved the sensitivity when compared with the AAB panel alone (93.5% vs. 67.5%; *P* < 0.0001) or with the Mayo model alone (93.5% vs. 63.6%; *P* < 0.0001) (Figure 3(d)); however, this combination could not improve the positive predictive values (PPVs) and the specificity when compared with either the panel alone or the model alone (Figures 3(e) and 3(f)).

### 3.5. The Performance of the 7-AAB Panel in combination with the Mayo Model in Distinguishing Benign from Advanced-Stage MPN

**Figure 4:**
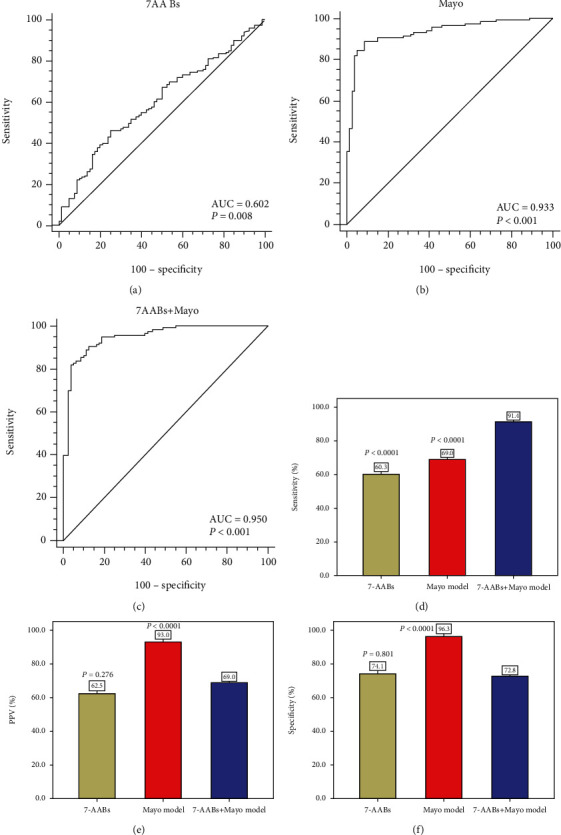
Diagnostic performance of the 7-AAB panel and the Mayo model between benign pulmonary nodules (BPN) and malignant pulmonary nodules (MPN) with advanced stage. (a) The AUC of the 7-AAB panel; (b) the AUC of the Mayo model; (c) the AUC of the 7-AAB panel combination with the Mayo model; (d) sensitivity in MPN patients with advanced stage; (e) positive predictive values (PPVs) in MPN patients with advanced stage; (f) specificity in BPN patients.

Next, we also evaluated the performance of the 7-AAB panel and the Mayo model in distinguishing advanced-stage (III-IV) MPN patients from the BPN controls. The 7-AAB panels of 116 advanced-stage MPN patients were measured, and their malignancy probabilities were calculated by the Mayo model. Advanced-stage MPN showed more patients with a nodule size > 8 mm and a malignancy probability > 65% compared to BPN. The AUCs (95% CI) for each model were as follows (Figures 4(a)–4(c)): the 7-AAB panel, 0.602 (0.536-0.665); the Mayo model, 0.933 (0.889-0.964); and the 7-AAB panel+the Mayo model, 0.950 (0.909-0.976). The diagnostic efficacy of the 7-AAB panel in combination with the Mayo model was better than that of the 7-AAB panel alone or the Mayo model alone. When the cutoff value of malignancy probability was set at 65%, the detection rate for advanced-stage MPN was 69.0%. The 7-AAB panel in combination with the Mayo model also significantly improved the sensitivity when compared with the 7-AAB panel alone (91.4% vs. 60.3%; *P* < 0.0001) or with the Mayo model alone (91.4% vs. 69.0%; *P* < 0.0001) (Figure 4(d)). However, this combination decreased the PPV and the specificity when compared with the Mayo model alone (69.0% vs. 93.0%, *P* < 0.0001; 72.8% vs. 96.3%, *P* < 0.0001, respectively) (Figures 4(e) and 4(f)).

## 4. Discussion

Identifying MPN is crucial in the early detection of LC. In this study, we incorporated a 7-AAB panel with the Mayo prediction model in the differential diagnosis of pulmonary nodules and early detection of lung cancer. Our results confirmed the clinical value of this 7-AAB panel in aiding the diagnosis of early-stage lung cancer, as the detection rate was superior to that of traditional tumor biomarkers. We also validated that the 7-AAB panel in combination with the Mayo model significantly increased the sensitivity, but the PPV and specificity could not be improved in comparison with the 7-AAB panel alone or the Mayo model alone in the differential diagnosis of MPN from BPN, whether MPN was at an early or advanced stage. Based on these findings, we suggest that the 7-AAB panel can be used as a biomarker for the early detection of lung cancer and that it can be incorporated with the Mayo model to determine the probability of malignancy of pulmonary nodules.

Novel biomarkers have been discovered and developed for use in early-stage LC screening, such as autoantibody panels, circulating microRNAs—especially small noncoding RNAs (ncRNAs), circulating tumor DNA, DNA methylation, complement fragments, blood protein profiles, or plasma lipid markers from lipidomics [30, 31, 32]. Among these, the autoantibody panel EarlyCDT-Lung has been reported and validated as an aid for the early detection of lung cancer [18, 19]. Many studies have investigated the diagnostic value of joint detection with AABs. Our review previously meta-analyzed four studies that measured the EarlyCDT-Lung Test 7-AAB panel (p53, CAGE, NYESO-1, GBU4-5, SOX2, MAGE A4, and Hu-D), showing a sensitivity of 47% (95% CI 0.34–0.60) with a high specificity of 90% (95% CI 0.87–0.93) in the early detection of lung cancer [19, 21, 33, 34, 35]. In the present study, we investigated a different 7-AAB panel (p53, GAGE7, PGP9.5, CAGE, MAGEA1, SOX2, and GBU4-5), which identified 67.5% of early-stage LC with a specificity of 89.6%. Although these 7-AAB panels possess high specificity as serum diagnostic markers in the diagnosis of early-stage lung cancer, the low sensitivity limits the application of the AAB panels in clinical practice. Thus, there is an urgent need to find approaches that can improve the sensitivity of the detection efficacy of early-stage LC. As noted previously, one recent study [13] evaluated the combination of a 7-AAB panel and low-dose computed tomography (CT) scanning and significantly improved the diagnostic yield in early-stage MPN patients, with the PPV significantly improving to 95.0% when compared with the AAB panel alone (95.0% vs. 85.2%; *P* < 0.001) or with CT scanning alone (95.0% vs. 69.0%; *P* < 0.001). Another study also found that this 7-AAB panel could distinguish malignant lesions from benign lesions and control cases, with a sensitivity of 56.53% and a specificity of 91.60%, but the specificity could be further increased to 95.80% when combined with CT [22]. To overcome the drawbacks of CT's high false-positive rate and radiologist subjectivity, in the present study, we combined a 7-AAB panel with the Mayo prediction model in the differential diagnosis of pulmonary nodules. The 7-AAB panel showed a sensitivity of 67.5% in the detection of early-stage MPN. However, the 7-AAB panel combined with the Mayo model had a significantly improved detection efficacy when compared with the AAB panel alone or the Mayo model alone, with sensitivities of 93.5% and 91.4% in distinguishing early- and advanced-stage malignant nodules, respectively, from benign nodules, but the PPV and specificity did not improve correspondingly. The results were not consistent with two previous studies that combined this 7-AAB panel with CT scans. [13, 22] We hypothesize that this could be related to the high false-positive rate of and subjective diagnosis from CT. For improving sensitivity, we view the 7-AAB panel and the Mayo model as complementary rather than competitive, and the combination of the two methods may be beneficial in distinguishing benign from malignant lesions, particularly early-stage MPN, which is potentially curable when detected early.

Recently, several prediction models including clinical and radiological values have been developed that can help physicians distinguish between benign and malignant nodules [9]. A study found that the Mayo, Veterans Association (VA), and Brock models showed similar predictive performance for malignant nodules (AUC: 0.6145, 0.6042, and 0.6820, respectively) and outperform the Herder model (AUC: 0.5567), which includes the [18]FDG uptake value [36]. Another study evaluated three prediction models, the Mayo, VA, and Peking University (PU) models. The area under the ROC curve of the PU model [0.800; 95% confidence interval (CI): 0.708-0.891] was higher than that of the Mayo model (0.753; 95% CI: 0.650-0.857) and of the VA model (0.728; 95% CI: 0.6230-833); however, these findings were not statistically significant. This means that these mathematical prediction models have similar accuracy for the prediction of SPN malignancy [11]. Therefore, we selected the most extensively validated Mayo model to aid in distinguishing between benign and malignant nodules. Moreover, our study applied the Mayo model to separately investigate early- or advanced-stage MPN patients, and the results showed that the model's AUC was 0.670 (95% CI: 0.605-0.730) for early-stage MPN and 0.933 (95% CI: 0.889-0.964) for advanced-stage MPN, which is in line with the recently reported literature [11, 36]. It seems that the Mayo model has greater accuracy for predicting malignant PNs at the advanced stage than at the early stage. We assume that this may be related to the larger nodule size and higher malignancy probability (>65%) in late-stage MPN. However, when the Mayo model was combined with the 7-AAB panel, the AUCs were significantly improved for both early-stage and advanced-stage MPN (0.795 and 0.950, respectively).

Because traditional TAA markers, such as cytokeratin 19 fragment antigen (CYFRA21-1), neuron-specific enolase (NSE), carcinoembryonic antigen (CEA), and squamous cell carcinoma antigen (SCC), remain widely used as reference diagnostics for lung cancer [37], we compared the 7-AAB panel with the combination of these traditional TAAs in the diagnosis of LC. We found that the 7-AAB panel was good in the early stages of lung cancer, while the traditional tumor markers showed a higher sensitivity with late-stage LC. This suggests that the 7-AAB panel is not suited for use as a biomarker for late-stage LC patients, whereas the traditional antigen biomarkers such as CEA, NSE, SCC, and CYFRA 21-1 should not be used to diagnose early-stage LC patients.

Identifying malignant pulmonary nodules and achieving the early detection of lung cancer significantly improve the survival rate and decreases mortality associated with this disease. Currently, the EarlyCDT-Lung test is being evaluated in a large-scale screening study in individuals at high risk of lung cancer worldwide. In addition to validating the clinical efficacy of the 7-AAB panel for the early detection of lung cancer, we also found that the combination of the 7-AAB panel with the Mayo model could significantly improve the sensitivity for distinguishing benign from malignant lesions at both early and late stages. As the ELISA of AABs is relatively low cost, noninvasive, and easy-to-perform, and the Mayo model is defined by equations, a combination based on the 7-AAB panel illustrated here and the Mayo model holds promise for the early detection of MPN, and it can be applied in some undeveloped areas or hospitals without high-resolution CT scans. Early detection of malignancy and timely resection are important while the nodule is still relatively small, as this could lead to decreased mortality.

Inevitably, there are some limitations in our research. First, the number of stage I-II MPN cases may not be sufficient. The BPN group was not matched well with the MPN groups for age, gender, or smoking status. Additionally, the study included no other cancer control group aside from lung cancer that could have had some or all AABs in common with those of the panel. Additionally, although the Mayo model was designed for pulmonary nodules, we did not investigate the AAB panel and the Mayo model with different sizes of PNs or subtypes of MPN to further evaluate their validity. Furthermore, we only analyzed the diagnostic efficacy of the AAB panel and the Mayo model in a Chinese population, and a future work is ongoing to validate the sensitivity and specificity of the combination in other ethnicities.

In conclusion, our study confirmed the clinical value of the 7-AAB panel for the early detection of lung cancer, which achieved a sensitivity of 67.5% and a specificity of 89.6%. This 7-AAB panel proved to be better than traditional tumor markers, such as CEA, NSE, SCC, and CYFRA 21-1, in aiding with early diagnosis. The combination of the 7-AAB panel with the Mayo model can improve the sensitivity for distinguishing benign PNs from malignant nodules, but the combination could not improve the PPV or the specificity. Taken together, this study illustrates the robust potential of the 7-AAB panel for the early diagnosis of lung cancer and in combination with the Mayo model could be used to distinguish the probability of malignancy of pulmonary nodules in clinical practice.

## Figures and Tables

**Table 1:** Clinical characteristics of the LC patients and controls.

Parameters	LC (*n* = 193)	BLD (*n* = 118)	HC (*n* = 135)	*P* value
Age (year)				
Range	28-82	35-87	28-87	
Mean (SD)	58.8 (9.9)	57.9 (10.6)	52.3 (11.2)	0.364
Gender				
Male	141 (73.1)	84 (71.2)	97 (71.9)	0.933
Female	52 (26.9)	34 (28.8)	38 (281)	
Smoking, *n* (%)				
Ever/current	128 (66.3)	72 (61.0)	73 (54.1)	0.081
Never	65 (33.7)	46 (39.0)	62 (45.9)	
7-AABs, *n* (%)				
Positive	122 (63.2)	20 (16.9)*^♀^*	14 (10.4)	<0.0001
Negative	71 (36.8)	98 (83.1)*^♀^*	121 (89.6)	
Cancer stage, *n* (%)		Diseases (*n*)		
I	32 (16.6)	Bronchitis (26)		
II	45 (23.3)	CAP (55)		
III	47 (24.4)	COPD (8)		
IV	69 (35.7)	Bronchiectasis (12)		
Cancer subtype, *n* (%)		Pulmonary tuberculosis (6)		
Adenocarcinoma	112 (58.0)	Parapneumonic effusion (4)		
Squamous cell carcinoma	39 (20.3)	OSAS (4)		
Large cell lung carcinoma	2 (1.0)	CVA (3)		
SCLC	40 (20.7)			

HC = health controls; LC = lung cancer; BLD = benign lung diseases; SD = standard deviation; SCLC = small cell lung cancer; COPD = chronic obstructive pulmonary disease; CAP = community-acquired pneumonia; CVA = cough-variant asthma; OSAHS = obstructive sleep apnea syndrome. Compared to HC, *^♀^*>0.05.

**Table 2:** Baseline characteristics and performances of the patients with PN.

Parameters	Advanced stage MPN (*n* = 116)	Early-stage MPN (*n* = 77)	BPN (*n* = 162)	*P* value
Age, median (range)	61.4 (40.0-82.0)^∗^	54.2^♀^ (28.0-77.0)	51.1 (29.0-75.0)	<0.01
Sex, *n* (%)				
Male	94 (81.0)^∗∗∗^	47 (61.0)^♀^	86 (53.1)	<0.0001
Female	22 (19.0)	30 (39.0)	76 (46.9)	
Smoking, *n* (%)				
Ever/current	86 (74.1)^∗∗∗^	42 (54.5)^∗∗∗^	49 (30.2)	<0.0001
Never	30 (25.9)	35 (45.5)	113 (69.8)	
Nodule size, *n* (%)				
≤8 mm	10 (8.6)^∗∗∗^	31(40.3)^∗∗∗^	134 (83.8)	<0.0001
9 mm-30 mm	28 (24.2)^∗^	45 (58.4)^∗∗∗^	20 (12.3)	<0.0001
>30 mm	78 (67.2)^∗∗∗^	1 (1.3)^♀^	8 (4.9)	<0.0001
7-AABs, *n* (%)				
Positive	70 (60.3)^∗∗∗^	52 (67.5)^∗∗∗^	42 (25.9)	<0.0001
Negative	46 (39.7)	25 (32.5)	120 (74.1)	
Mayo model, *n* (%)				
<5%	4 (3.4)^∗∗∗^	28 (36.4)^∗∗∗^	100 (61.7)	<0.0001
5–65%	32 (27.6)^♀^	42 (54.5)^∗∗^	56 (34.6)	0.001
>65%	80 (69.0)^∗∗∗^	7 (9.1)^♀^	6 (3.7)	<0.0001

PN = pulmonary nodule; MPN = malignant pulmonary nodule; BPN = benign pulmonary nodule. Compared to BPN, ^∗^<0.05, ^∗∗^<0.001, ^∗∗∗^<0.0001, and ^♀^>0.05.

## Data Availability

Data is available on request. Due to China's new laws and regulations on biosafety data control, the original data protecting patient information is under control. If you need raw data, you can apply with the corresponding author (huzhuojun1964@163.com).

## Abbreviations

LC: Lung cancer
MPN: Malignant pulmonary nodule
ELISA: Enzyme-linked immunosorbent assay
BPN: Benign pulmonary nodule
PN: Pulmonary nodule
SPN: Solitary pulmonary nodule
AAB: Autoantibody
GGN: Ground-glass nodule
BLD: Benign lung diseases
NSCL: Non-small-cell lung cancer
SCLC: Small-cell lung cancer
ROC: Receiver operating characteristic
AUC: Area under the curve
CT: Computed tomography
LDCT: Low dose computed tomography
TAA: Tumor-associated antigen
TM: Tumor marker
SD: Standard deviation
PPV: Positive predictive value
CAP: Community-acquired pneumonia
COPD: Chronic obstructive pulmonary disease
CVA: Cough-variant asthma
OSAS: Obstructive sleep apnea syndrome.
